# Clinical characteristics and management of dasatinib-induced chylothorax: retrospective analysis based on case reports

**DOI:** 10.3389/fphar.2025.1665484

**Published:** 2025-09-29

**Authors:** Ya Liu, Ying Huang, Jincai Guo, Xiang Liu, Yixiang Hu

**Affiliations:** ^1^ Department of Clinical Pharmacy, Xiangtan Central Hospital (The Affiliated Hospital of Hunan University), Xiangtan, China; ^2^ Department of Respiratory Medicine, Zhongshan Hospital of Traditional Chinese Medicine Afflilated to Guangzhou University of Chinese Medicine, Zhongshan, China; ^3^ Department of Pharmacy, Changsha Stomatological Hospital, Changsha, China; ^4^ State Key Laboratory of Chemo/Bio-Sensing and Chemometrics, School of Biomedical Sciences, Hunan University, Changsha, China

**Keywords:** dasatinib, chylothorax, chronic myeloid leukemia, pleural effusion, diagnosis

## Abstract

**Background:**

Dasatinib-induced chylothorax is a rare but potentially serious complication, and its clinical features remain poorly defined. This study aimed to systematically evaluate its clinical characteristics, therapeutic approaches, and patient outcomes to provide practical guidance for clinical management.

**Methods:**

We conducted a retrospective analysis by systematically retrieving case reports of dasatinib-induced chylothorax from relevant databases up to 31 May 2025, to achieve a comprehensive assessment.

**Results:**

A total of 24 patients from 22 published case reports and case series were included in this analysis. The median age was 50 years (range 5, 79), and the median time to onset of chylothorax following dasatinib initiation was 41 months (range 1, 168). The most frequently reported clinical symptom was dyspnea (62.5%), followed by fever (25.0%), cough (16.7%), abdominal pain (8.3%), and chest pain (4.2%). Pleural fluid was typically described as white and milky in appearance (87.5%). Biochemical analysis revealed a median pleural fluid triglyceride concentration of 547.4 mg/dL and an albumin level of 4.4 g/dL. Dasatinib therapy was discontinued in 83.3% of patients. Alternative tyrosine kinase inhibitors were prescribed in selected cases, including nilotinib (33.3%), imatinib (20.8%), and bosutinib (16.7%). Supportive treatments consisted primarily of corticosteroids (45.8%), diuretics (45.8%), and thoracentesis (29.2%). Clinical improvement was observed in 91.7% of patients, with a median time to recovery of 12.2 weeks (range 1, 52). Among the four patients who underwent dasatinib rechallenge, three experienced recurrence of chylothorax.

**Conclusion:**

Dasatinib-induced chylothorax is rare but often reversible. Early diagnosis, drug discontinuation, and appropriate supportive care are key to recovery. Rechallenge should be undertaken cautiously due to a high risk of recurrence.

## Introduction

Dasatinib, an oral second-generation BCR-ABL tyrosine kinase inhibitor (TKI), received approval from the U.S. Food and Drug Administration in 2006 for patients with Philadelphia chromosome-positive (Ph+) chronic myeloid leukemia (CML) or acute lymphoblastic leukemia (ALL) who were resistant or intolerant to prior therapy, and was subsequently approved in 2010 for newly diagnosed chronic-phase CML (Foà et al., 2024; Advani et al., 2023). Compared with the first-generation TKI imatinib, dasatinib exhibits greater potency and broader kinase inhibition, targeting not only BCR-ABL but also a spectrum of kinases including Src-family kinases (SFKs), KIT proto-oncogene receptor tyrosine kinase (c-KIT), platelet-derived growth factor receptor beta (PDGFR-β), and ephrin type-A receptor 2 (EPHA2) (Keating, 2017). This expanded inhibitory profile underlies its superior therapeutic efficacy while also accounting for distinct adverse-event patterns. While adverse events are generally infrequent, pleural effusion has emerged as a notable complication, and in rare instances, this may evolve into chylothorax—an accumulation of chyle in the pleural space, typically presenting with milky effusion rich in triglycerides and lymphocytes (Huang et al., 2023). The underlying pathophysiology of dasatinib-induced chylothorax is not fully understood, but it is thought to involve off-target effects on lymphatic vessels and alterations in vascular permeability (Molina et al., 2020). To date, available data on dasatinib-induced chylothorax are primarily from case reports and small series, providing limited guidance for clinicians in terms of diagnosis and management. Understanding the clinical features, diagnostic strategies, and treatment options is crucial for improving patient outcomes. This study aims to retrospectively analyze the clinical characteristics, management approaches, and outcomes of patients with dasatinib-induced chylothorax, offering insights that may aid in the early recognition and effective management of this rare complication.

## Methods

### Study design and data collection

A systematic search was conducted in PubMed, EMBASE, Web of Science, and Chinese databases (WanFang, CNKI) for studies published up to 31 May 2025. The search used a combination of keywords: “Dasatinib” OR “Tyrosine kinase inhibitor” OR “Src-family kinases” AND “Chylothorax” OR “Pleural effusion” OR “pleural fluid” OR “chyle”. Only case reports, case series, and clinical studies reporting on dasatinib-induced chylothorax with detailed clinical data, were included. Duplicate studies were removed, and references from eligible articles were reviewed for additional relevant studies.

### Inclusion and exclusion criteria

We included clinical studies, case reports, and case series that (1) reported patients with dasatinib-induced chylothorax (Advani et al., 2023), provided comprehensive clinical data (e.g., demographic details, clinical presentation, pleural fluid analysis, treatment, and outcomes). Exclusion criteria were reviews, mechanism studies, animal studies, duplicate cases, and articles with insufficient data.

## Data extraction

A standardized form was used to extract relevant information from each eligible article. The following data were collected: patient characteristics (age, sex), underlying disease (CML, Ph + ALL, etc.), dasatinib dosage and treatment duration, time to onset of chylothorax, presenting symptoms, imaging findings, pleural fluid characteristics (triglycerides, lymphocytes, albumin, lactate dehydrogenase), management strategies (discontinuation of dasatinib, use of alternative TKIs, supportive treatments), and patient outcomes (recovery, recurrence, or mortality).

### Causality assessment

Causality was evaluated based on the WHO-UMC system, which classifies cases as “certain,” “probable,” “possible,” or “unlikely.” A “certain” classification was given when the onset was temporally related to dasatinib use, symptoms improved after discontinuation, and recurrence occurred upon rechallenge. “probable” cases had a reasonable temporal link, with improvement upon discontinuation but without rechallenge. “possible” cases had a reasonable temporal association, but other factors could not be ruled out. “unlikely” cases were those where the temporal relationship with dasatinib was unclear, and other explanations for the adverse event were more likely.

### Statistical analysis

Descriptive statistics were used to summarize the data. Continuous variables were expressed as medians with ranges (minimum to maximum), while categorical variables were presented as percentages. All analyses were performed using SPSS version 25.0.

## Results

### Basic information

**FIGURE 1:**
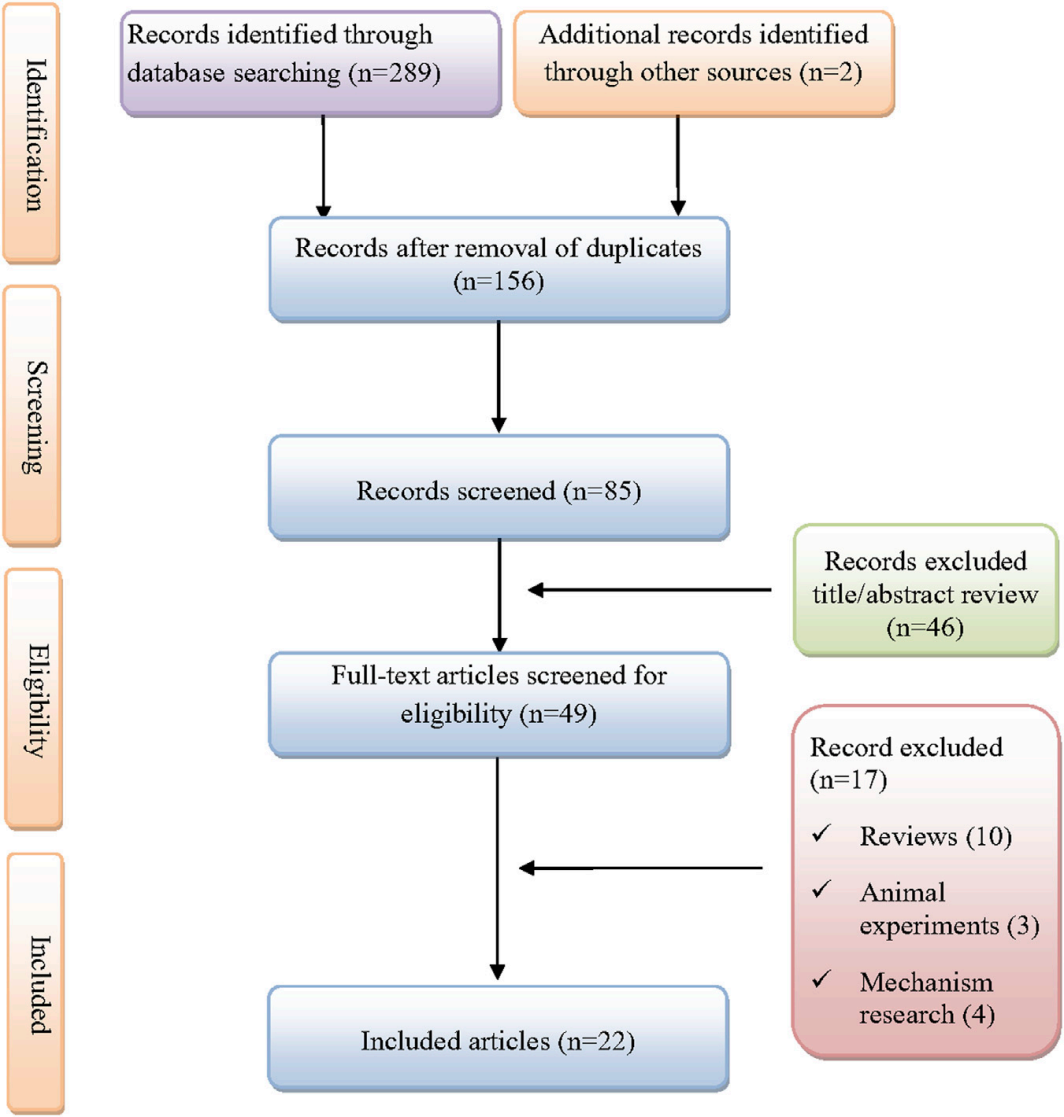
Flow diagram of the selection of studies for inclusion.

As shown in Figure 1, A total of 291 records were initially identified through database searches and manual screening. After removing duplicates and screening titles and abstracts, 22 studies were included for final analysis (Molina et al., 2020; Al-Abcha et al., 2019; Alqattan et al., 2022; Baloch et al., 2017; Bradt et al., 2022; Chen et al., 2020; Ferreiro et al., 2016; Garcia-Zamalloa et al., 2023; Hickman et al., 2020; Hsu et al., 2021; Huang et al., 2015; Justin et al., 2025; Kelly et al., 2022; Liu et al., 2023; Makimoto et al., 2022; Martínez et al., 2023; McGonegal and Baskar, 2024; Ng et al., 2024; Pai and Huang, 2025; Paul et al., 2021; Sasaki et al., 2019). A total of 24 patients with dasatinib-induced chylothorax were included in the analysis (Table 1). The median age was 50 years, ranging from 5 to 79 years, with a male predominance (58.3%). Most cases were reported from China (33.3%), followed by the United States (16.7%), Spain (12.5%), Singapore (8.3%), Japan (8.3%), Belgium (4.2%), Kuwait (4.2%), and Qatar (4.2%). The median time from dasatinib initiation to symptom onset was 41 months (range 1, 168), with over half of the patients (54.2%) developing symptoms within the first 30 months of therapy. The most common indication for dasatinib use was chronic myeloid leukemia (87.5%), followed by mixed phenotype acute leukemia (6.7%) and acute lymphoblastic leukemia (4.2%). Comorbidities were present in 58.3% of patients and included conditions such as chronic obstructive pulmonary disease, hypothyroidism, asthma, breast cancer, heart failure, and intolerance to imatinib or nilotinib. Among the 22 patients with reported dosing information, the most common dasatinib dose was 100 mg daily (59.2%), followed by 50 mg (18.2%), and other doses ranging from 60 mg to 140 mg. Concomitant medications were reported in three patients, including eltroxin, tacrolimus, methotrexate, ceftriaxone, and remdesivir.

**TABLE 1:** General data of 24 patients reported in case series/reports.

Parameter	Classification	Value
Gender (24)^a^	Male	14 (58.3%)
	Female	10 (41.7%)
Age (24)^a^	Years	50 (5, 79)^b^
Country (24)^a^	China	8 (33.3%)
	United States	5 (16.7%)
	Spain	4 (12.5%)
	Singapor	2 (8.3%)
	Japan	2 (8.3%)
	Belgium	1 (4.2%)
	Kuwait	1 (4.2%)
	Qatar	1 (4.2%)
Symptom onset time (24)^a^	Months	41 (1, 168)^b^
	1–30	13 (54.2%)
	31–60	6 (25.0%)
	61–90	1 (4.2%)
	91–120	2 (8.3%)
	121–168	2 (8.3%)
Indication (24)^a^	Chronic myeloid leukemia	21 (87.5%)
	Acute lymphoblastic leukemia	1 (4.2%)
	Mixed phenotype acute leukemia	2 (6.7%)
Disease history (14)^a^	COPD, hypothyroidism, breast cancer, asthma, imatinib and nilotinib intolerant, heart failure	14 (58.3%)
Dasatinib dosage (22)^a^	50 mg daily	4 (18.2%)
	60 mg daily	1 (4.5%)
	70 mg daily	1 (4.5%)
	90 mg daily	1 (4.5%)
	100 mg daily	13 (59.2%)
	140 mg daily	2 (9.1%)
Concomitant medications (3)^a^	Eltroxin	1 (33.3%)
	Tacrolimus and methotrexate	1 (33.3%)
	Ceftriaxone and remdesivir	1 (33.3%)

^a^ Represents the number of patients with this parameter out of 24 patients.

^b^ Median (minimum, maximum).

### Clinical manifestations

Respiratory symptoms were the most prominent clinical presentations of dasatinib-induced chylothorax (Table 2). Dyspnea was reported in 62.5% of patients, followed by fever (25.0%) and cough (16.7%). Less frequent symptoms included abdominal pain and hypoxia (8.3% each), as well as chest pain and pulmonary hypertension (4.2% each). Imaging findings revealed bilateral pleural effusions in 45.8% of patients, while right-sided and left-sided effusions were observed in 29.2% and 25.0%, respectively. Additional radiological abnormalities included atelectasis (8.3%), pericardial effusion (4.2%), ascites (4.2%), and cardiomegaly (4.2%). According to the WHO-UMC system, 12.5% of cases were classified as “certain,” 70.8% as “probable,” and 16.7% as “possible” (see Supplementary Data Table 1).

**TABLE 2:** Clinical information of 24 included patients.

Parameter	Classification	Value
Clinical symptoms (24)^a^	Dyspnea	15 (62.5%)
	Fever	6 (25.0%)
	Cough	4 (16.7%)
	Abdominal pain	2 (8.3%)
	Hypoxia	2 (8.3%)
	Chest pain	1 (4.2%)
	Pulmonary hypertension	1 (4.2%)
Chest imaging examination (24)^a^	Bilateral pleural effusion	11 (45.8%)
	Right-sided pleural effusion	7 (29.2%)
	Left-sided pleural effusion	6 (25.0%)
	Atelectasis	2 8.3%)
	Pericardial effusion	1 (4.2%)
	Ascites	1 (4.2%)
	Cardiomegaly	1 (4.2%)
Thoracentesis and fluid analysis (24)^a^	White milky fluid	21 (87.5%)
	Bloody milky fluid	2 (8.3%)
	Yellow milky fluid	1 (4.2%)
Pleural fluid triglyceride (21)^a^	mg/dL	547.4 (110, 1374)^b^
Pleural fluid Albumin (14)^a^	g/dL	4.4 (3.3, 6.0)^b^
Pleural fluid lactate dehydrogenase (17)^a^	U/L	179.2 (99, 349)^b^
Pleural fluid cholesterol (2)^a^	mg/dL	189.5 (106, 273)^b^
Pleural fluid lymphocyte (13)^a^	%	86.1 (56, 98.8)^b^
Serum triglyceride (7)^a^	mg/dL	289.3 (80, 685)^b^
Serum albumin (6)^a^	g/dL	3.3 (2.3, 4.4)^b^
WHO-UMC causality category (24)^c^	Certain	3 (12.5%)
	Probable	17 (70.8%)
	Possible	4 (16.7%)

^a^ Represents the number of patients with this parameter out of 24 patients.

^b^ Median (minimum, maximum).

^c^ Under the WHO-UMC, system, a “certain” case shows a clear temporal relationship with drug use, improvement upon withdrawal, and recurrence upon rechallenge. A “probable” case has a reasonable time relationship, is unlikely explained by other causes, and improves after withdrawal without requiring rechallenge. A “possible” case has a reasonable time relationship but may also be due to other conditions, and the effect of withdrawal is unclear.

Chylothorax defined as pleural TG > 110 mg/dL (>1.24 mmol/L) or chylomicrons with lymphocyte predominance >70%. Serum reference ranges: TG < 150 mg/dL (<1.7 mmol/L); albumin 3.5–5.0 g/dL.

### Thoracentesis and fluid analysis

All patients underwent thoracentesis. Pleural findings were interpreted against standard thresholds for chylothorax—pleural triglycerides (TG) > 110 mg/dL (>1.24 mmol/L) or chylomicrons present, with lymphocyte predominance >70% supporting the diagnosis. As summarized in Table 2, the pleural fluid was predominantly white and milky in appearance (87.5%), consistent with chylous effusion, while a smaller proportion appeared bloody milky (8.3%) or yellow milky (4.2%). Biochemically, pleural TG was markedly elevated, with a median of 547.4 mg/dL and a range of 110–1374 mg/dL, and the differential showed lymphocyte predominance (median 86.1%, range 56.0%–98.8%). The median pleural fluid albumin level was 4.4 g/dL, and lactate dehydrogenase (LDH) had a median value of 179.2 U/L. For serum measurements, we applied widely recognized reference ranges (triglycerides <150 mg/dL [<1.7 mmol/L]; albumin 3.5–5.0 g/dL) to aid interpretation. Among cases with available data, the median serum triglyceride level was 289.3 mg/dL (3.27 mmol/L) and serum albumin was 3.3 g/dL, indicating hypertriglyceridemia with mild hypoalbuminemia.

### Treatment and prognosis

As shown in Table 3, following the diagnosis of chylothorax, dasatinib was discontinued in most cases (83.3%, n = 20). Among these, 33.3% of patients (n = 8) were switched to nilotinib, 20.8% (n = 5) to imatinib, and 16.7% (n = 4) to bosutinib as alternative tyrosine kinase inhibitors. Supportive treatment was commonly administered and included corticosteroids and diuretics in 45.8% of patients (n = 11 each), while thoracentesis or pleural drainage was performed in 29.2% (n = 7). Octreotide was used in two cases (8.3%). Clinical improvement was achieved in 91.7% of patients (n = 22). Among the 15 patients with available recovery time data, the median time to resolution was 12.2 weeks (range: 1–52). Most patients (66.7%, n = 11) recovered within the first 10 weeks, while a smaller proportion required prolonged treatment duration, with recovery extending up to 52 weeks in isolated cases. Rechallenge with dasatinib was attempted in four patients, of whom three (75.0%) experienced recurrence of chylothorax, highlighting a high risk of relapse upon re-exposure.

**TABLE 3:** Treatment and prognosis of 24 patients reported in case series/reports.

Parameter	Classification	Value
Treatment (24)^a^	Discontinuation	20 (83.3%)
	Switch to nilotinib	8 (33.3%)
	Switch to imatinib	5 (20.8%)
	Switch to bosutinib	4 (16.7%)
	Steroids	11 (45.8%)
	Diuretics	11 (45.8%)
	Thoracentesis/drainage	7 (29.2%)
	Octreotide	2 (8.3%)
Outcome (24)^a^	Improvement	22 (91.7%%)
	No recovery	2 (8.3%)
Recovery time (15)^a^	Weeks	12.2 (1, 52)^b^
	1–10	11 (66.7%)
	11–20	1 (6.7%)
	21–30	2 (13.3%)
	31–40	1 (6.7%)
	41–52	1 (6.7%)
Rechallenge (4)^a^	Relapse	3 (75%)
	No recurrence	1 (25%)

^a^ Represents the number of patients out of 30 in whom information regarding this particular parameter was provided.

^b^ Median (minimum, maximum).

## Discussion

Chylothorax primarily occurs following disruption of the thoracic duct, which may result from traumatic injury such as cardiac surgery, or from non-traumatic causes including lymphatic obstruction associated with sarcoidosis, lymphoma, or superior vena cava syndrome (Armas-Villalba and Jimenez, 2025). In contrast, pleural effusion is a far more common non-hematologic toxicity of dasatinib, occurring in 20%–30% of CML patients, often within the first year of therapy, and is associated with immune-mediated mechanisms, endothelial permeability changes, and fluid retention (Breccia et al., 2011; Jain et al., 2024). Chylothorax, however, is exceedingly rare and generally develops after prolonged exposure. Eskazan et al. reported that dasatinib-related pleural effusion may be accompanied by pericardial effusion but rarely by ascites, suggesting selective serosal involvement (Küçükyurt et al., 2024). These findings highlight chylothorax as a rare and distinct complication of dasatinib, with timing, mechanisms, and clinical implications differing from pleural effusion. In clinical practice, diagnostic confirmation of chylothorax is based on pleural fluid characteristics, which often present as a milky effusion with elevated triglycerides and predominance of lymphocytes. Widely accepted criteria include triglyceride levels >110 mg/dL (>1.24 mmol/L) or the presence of chylomicrons, with nucleated cell counts showing >70% lymphocytes further supporting a chylous etiology (Armas-Villalba and Jimenez, 2025; Riley and Ataya, 2019). While medication-induced chylothorax is rare, dasatinib is recognized as an uncommon pharmacological cause, representing one of the few drug-related etiologies identified to date (Agrawal et al., 2022). As noted in our analysis, the majority of patients developed chylothorax after prolonged exposure to dasatinib, with the median time to symptom onset being 41 months. This finding indicating that chylothorax typically arises in the later stages of anti-myeloid leukemia treatment following extended drug exposure. Noteworthy, reported cases span a broad age range, from pediatric to elderly patients, underscoring the need for vigilance across age groups. Given the limited pediatric evidence, decisions regarding dose adjustment, switching to alternative TKIs, and the appropriateness of rechallenge should be individualized and carefully balanced against the risk of recurrence. Although the exact pathophysiological mechanisms remain unclear, they are thought to involve dasatinib-induced disruption of lymphatic function and vascular integrity, potentially through the inhibition of PDGFR-β and SFKs, both of which are crucial for maintaining lymphatic drainage and vascular stability (Huang et al., 2015; Bergers et al., 2003). Mechanistically, PDGFR-β regulates lymphangiogenesis, and its inhibition leads to the formation of abnormal lymphatic vessels, resulting in leakage into the pleural space (Zhang et al., 2024). Similarly, SFKs inhibition can modulate endothelial barrier function and increase vascular permeability, potentially destabilizing the pleural microvasculature and favoring effusion formation (Cai et al., 2020). However, the specific reasons behind dasatinib’s heightened affinity for the vasculature and lymphatics remain unclear.

Dasatinib has a half-life of approximately 3–5 h and is primarily metabolized by the liver via the CYP3A4 enzyme, with excretion occurring through bile and urine (Advani et al., 2023). The typical dosage of dasatinib is administered once daily, usually at doses of 50 mg or 100 mg, adjusted based on the patient’s specific condition and tolerance (Pontarollo and Reinhardt, 2023). Notably, dasatinib is associated with a broad adverse-event profile that includes hematologic toxicities (e.g., neutropenia, anemia, thrombocytopenia), cardiopulmonary events (e.g., pleural effusion, pulmonary arterial hypertension, arrhythmias), hepatic dysfunction, cutaneous reactions, and fluid-retention syndromes, as well as systemic symptoms such as headache and fatigue (Pontarollo and Reinhardt, 2023). In our analysis of 24 patients, the most commonly reported clinical manifestations were dyspnea (62.5%), followed by fever (25.0%), cough (16.7%), and abdominal pain and hypoxia. The diagnosis of dasatinib-induced chylothorax is confirmed through pleural fluid analysis, which typically shows a characteristic milky appearance and elevated triglyceride levels, findings that were observed in the majority of our cases. The pleural fluid analysis also showed a predominance of lymphocytes, which is consistent with the typical presentation of chylothorax. This highlights the critical role of pleural fluid analysis in differentiating dasatinib-induced chylothorax from other potential causes of pleural effusion, such as malignancy or infection. Given that this complication is relatively rare, clinicians must maintain a high degree of suspicion when faced with patients on dasatinib therapy presenting with pleural effusion.

Management of dasatinib-induced chylothorax primarily involves discontinuing the drug, which was associated with clinical improvement in the majority of patients in our analysis. Alternative TKIs such as nilotinib, imatinib, and bosutinib were frequently used in place of dasatinib, with no recurrence of chylothorax in most cases. Nilotinib is generally considered a suitable alternative owing to its efficacy and lower incidence of pleural effusion compared with dasatinib (Jain et al., 2024). Imatinib, although less potent, remains a well-tolerated option, particularly in patients with stable disease or those intolerant to other second-generation TKIs (Jain et al., 2024; Cortes et al., 2016; Paydas, 2014). Bosutinib, another second-generation TKI, has been associated with gastrointestinal toxicities but rarely with pleural or chylous effusions, making it a reasonable alternative in selected cases (Cortes et al., 2018). Clinicians should individualize the choice of an alternative TKI based on prior therapy, comorbidities, and overall risk-benefit assessment. In addition to drug discontinuation, supportive treatments, including corticosteroids, diuretics, and thoracentesis, were employed and were effective in most patients. These interventions contributed significantly to symptom resolution, with the median recovery time being 12.2 weeks. Although most patients recovered within 10 weeks, some required a prolonged treatment course, with recovery extending up to 52 weeks in a few isolated cases. Of particular concern is the high rate of recurrence of chylothorax upon rechallenge with dasatinib. Notably, three out of four patients who were rechallenged with dasatinib experienced a recurrence of symptoms, highlighting the high risk of relapse with re-exposure to the drug. This finding underscores the high risk of relapse with rechallenge and suggests that rechallenging with dasatinib should be avoided once chylothorax has developed. In refractory cases, additional therapeutic approaches have been described. For instance, a single case report documented the use of Goreisan combined with diuretics, which achieved control of dasatinib-induced chylothorax (Sasaki et al., 2019). Nevertheless, because of the concomitant treatment, the independent efficacy of Goreisan remains uncertain and warrants further evaluation.

### Limitations of the study

This study has several limitations that should be acknowledged. First, the analysis is based on a limited number of published case reports, which may lead to selection and publication bias. Secondly, incomplete clinical data in some reports, particularly the absence of prognostic information in certain patients, may limit the accuracy of the analysis. Third, the retrospective nature of the study precludes standardized data collection and limits the ability to evaluate causality or treatment efficacy in a controlled manner. Finally, due to variability in diagnostic approaches and management strategies across reports, it remains difficult to establish uniform recommendations. Nevertheless, this study summarizes current evidence and may aid clinicians in the early recognition and appropriate management of dasatinib-induced chylothorax.

## Conclusion

In conclusion, dasatinib-induced chylothorax is a rare but serious complication that requires early recognition and prompt management. Discontinuation of dasatinib, combined with appropriate supportive therapies, remains the cornerstone of treatment. The high risk of recurrence upon rechallenge underscores the need for caution when considering re-exposure to the drug. Further research is needed to explore alternative treatment options and to understand the underlying mechanisms of this complication.

## Data Availability

The original contributions presented in the study are included in the article/Supplementary Material, further inquiries can be directed to the corresponding authors.
